# Microsporidian obligate intracellular parasites subvert autophagy of infected mammalian cells to promote their own growth

**DOI:** 10.1128/mbio.01049-25

**Published:** 2025-05-30

**Authors:** Johan Panek, Eugenie Carriere, Moudy Bin Saleh, Kacper Sendra, Gregor Kosta, Viktor I. Korolchuk, Robert P. Hirt

**Affiliations:** 1 Biosciences Institute, Faculty of Medical Sciences, Newcastle University 12186, Newcastle upon Tyne, England, United Kingdom; 2 Laboratoire Microorganismes: Génome et Environnement, CNRS UMR 6023, Université Clermont Auvergne 27006, Clermont-Ferrand, Auvergne-Rhône-Alpes, France; 3 Department of Zoology, Faculty of Science, King Saud University 37850, Riyadh, Saudi Arabia; Albert Einstein College of Medicine, Bronx, New York, USA

**Keywords:** Microsporidia, intracellular parasite, autophagy, *Encephalitozoon cuniculi*, RK-13 cells, CACO-2 cells, gut mucosa

## Abstract

Intracellular pathogens such as Microsporidia can interfere with host proteostasis pathways, including autophagy. While the manipulation of host autophagy has been demonstrated in a nematode-infecting species, and autophagic activity was also observed in tardigrade midgut infections, it remains unclear whether this strategy extends to mammalian-infecting Microsporidia. Here, we investigated interactions between host autophagy and two human-pathogenic Microsporidian species representing distinct evolutionary lineages. Using immunochemistry, super-resolution fluorescence microscopy, and modulation of autophagy via siRNA silencing and chemical agents, we show that *Encephalitozoon cuniculi* is tagged by early autophagy markers (ubiquitin and p62) but escapes clearance via autolysosomes. Instead of restricting the parasite, autophagy induction significantly enhances Microsporidia proliferation in two mammalian cell models. Conversely, autophagy suppression—via siRNA or treatment with microbiota-derived metabolites important for gut epithelial homeostasis—reduces parasite growth. These findings demonstrate that the ability to evade and exploit host autophagy is not restricted to nematode-infecting species but is conserved across diverse Microsporidia infecting mammals. Together with adaptations such as NTT nucleotide transporters, the hijacking of autophagy emerges as a core strategy supporting the obligate intracellular lifestyle of these pathogens.

**IMPORTANCE:**

Microsporidia are tiny parasites that must live inside other cells to survive. In animals like worms and tardigrades, they’ve been seen to interact with a cell’s recycling system called autophagy, which usually helps the host defend itself. But what about Microsporidia that infect mammals, including humans? Our research shows that instead of being destroyed by autophagy, human-infecting Microsporidia use it to grow faster. We studied two different species in mammalian cells and found that when we boosted the host’s autophagy system, the parasites multiplied more. When we slowed down autophagy, parasite growth dropped. This means that Microsporidia have evolved clever ways to turn the host’s defences into a resource. Understanding how they do this could lead to better treatments for infections, especially for people with weakened immune systems. It also reveals a surprising twist in how these unusual parasites survive across a broad range of hosts.

## INTRODUCTION

Protein homeostasis is central to cellular physiology and ultimately to many aspects of health and disease in humans (1). Proteostasis relies on complex cellular regulatory networks that include two major interconnected proteolytic degradation pathways: the ubiquitin-proteasome system and the autophagy-lysosome (autophagy) pathway (2). Genetic predispositions, ageing, infectious agents, and abiotic environmental factors can all lead to dysregulation of these two pathways, which, in turn, underpins the pathobiology of numerous conditions. This includes infectious (e.g., HIV), neurodegenerative (e.g., Alzheimer’s disease), chronic inflammatory conditions (e.g., inflammatory bowel disease [IBD] and rheumatoid arthritis), metabolic syndrome-associated diseases, and cancers (3).

Because they are building their ecological niche inside the host cell, intracellular pathogens interact with host proteostasis, particularly with the autophagy pathway. Indeed, autophagy can play a dual role in the survival of intracellular pathogens by both maintaining nutrient balance of the host that can be exploited by the pathogen to multiply or by directly targeting intracellular pathogens for degradation in a pathway called xenophagy (4). As a result, several intracellular pathogens developed strategies to either escape this defence mechanism or subvert it. This is best documented for intracellular bacterial pathogens such as *Mycobacterium tuberculosis* or *Listeria* spp. that have developed specific mechanisms/virulent factors to interfere with the maturation of the autophagosome, or *Salmonella* spp. which secretes a deubiquitylation enzyme to remove its tagging by host ubiquitin and thus can evade xenophagy altogether (5, 6). In contrast, far less is known about the intracellular eukaryotic parasites and their interaction with host cell autophagy. However, their importance has been increasingly recognized in recent years, and studies have shown how some microbial eukaryotic parasites have developed strategies to escape and take advantage of autophagy. For example, during its liver stages, *Plasmodium* spp. is targeted by host cell xenophagy, as attested by rapid labelling of the meronts with host LC3 and SQSTM1/p62 proteins (7). However, this targeting does not lead to the engulfment of the parasite by canonical autophagosomes. Instead, it is located in the structure that will eventually become the parasitophorous vacuole (PV), suggesting an active recruitment of LC3 and a subversion of xenophagy by the parasite (8). This was confirmed by subsequent studies where induction of autophagy by either starvation or rapamycin treatment in mice increased the parasite load of *Plasmodium berghei* (9). Notably, in the same study, genetic suppression of autophagy through an *Atg5* knockout was both favouring an increase of pathogen load and reducing the size of the parasites, illustrating the dual role of autophagy in controlling the parasite and allowing it to divert nutrients from the host cell for its own benefit (9).

Microsporidia are a group of highly diverse strict obligate intracellular pathogens that infect most animal lineages, including economically and ecologically important species (10, 11). Several species can infect humans and represent a significant threat to immunocompromised patients (12). Recent studies have also shown that asymptomatic Microsporidia infections are more common in healthy immune-competent individuals than previously thought (13) and may be associated with chronic conditions including Crohn’s disease (CD) and cancer (14, 15). So far, only a few studies have investigated the interaction between Microsporidia and autophagy. In the tardigrade *Isohypsibius granulifer granulifer,* microsporidia infection was found by electron microscopy in the midgut, and the infected cells showed induced autophagic activity as well as xenophagy targeting the sporonts (16). More extensive studies were done in the *Caenorhabditis elegans* model (17, 18) where it was suggested that the Microsporidian species *Nematocida parisii* is targeted by *C. elegans* xenophagy machinery in a CUL-6-dependent manner, leading to the labelling of the parasite by host ubiquitin and LC3 (17). Knockdown of key autophagy genes was sufficient to produce a moderate but significant increase in the parasite load. All these results suggested that in the worm, autophagy plays a role in controlling the infection through the xenophagy pathway (17). In contrast, there is currently no such data in the mammalian systems. In mammals, including humans, the intestinal epithelium is the main site of Microsporidia infection (11). Perturbation of intestinal autophagy, which was shown to be a major cause of intestinal disorders such as IBDs or CD, was also linked to Microsporidia infection (19, 20). Therefore, investigating the interplay between mammalian cell autophagy and the Microsporidian species is of major interest.

We investigated this interplay using *Encephalitozoon cuniculi*, one of the most common Microsporidian species infecting mammals (21). This species was originally isolated from rabbits but is also commonly found in other mammals, including humans (22). The diminutive *E. cuniculi* genome is the result of massive genomic and protein coding capacity reduction (2.2 Mb encoding only 2,000 proteins) leading to the loss of multiple metabolic capabilities (21, 23, 24). These include the loss of numerous biosynthesis pathways and, notably, multiple genes mediating proteostasis, including the mechanistic Target of Rapamycin (mTOR) pathway as well as the majority of the autophagy machinery (25). This makes *E. cuniculi* an ideal model system to study the host autophagy response to an eukaryotic intracellular pathogen as it will not be impacted by mTOR inhibitors used to artificially modulate host cell autophagy. Previous transcriptomics analyses found that, similar to *C. elegans,* rabbit kidney cells (RK-13) infected with Microsporidia *T. hominis* (26) were characterised by an upregulation of genes encoding key autophagy components (27). While only *ATG4C* was found to be significantly upregulated in the original data set (log^2^ fold change: 1.59, adjusted *P* value: 0.0025), 10 other genes had increased expression upon infection, including several key *ATG* genes as well as 4 different Cullin genes, suggesting that autophagy/xenophagy may be stimulated upon infection in the mammalian system (26). Here, we exploited a combination of antibodies developed to investigate *E. cuniculi* infection cycle (28), pharmacological treatments, and genetic manipulation on mammalian cells to investigate the interplay between Microsporidia infection and mammalian autophagy.

## RESULTS

### *E. cuniculi* increases autophagy in mammalian cells but is only partially targeted by host cell autophagy

In previous transcriptomics analyses, we demonstrated that infection by *T. hominis* led to an upregulation of key components of the autophagy and ubiquitin-proteasome system in rabbit kidney (RK-13) cells, suggesting that autophagy/xenophagy might also be induced upon infection in mammalian cells (26). Since autophagy is largely regulated at the post-translational level, we aimed to validate these transcriptional findings by measuring autophagic flux in RK-13 cells infected with *E. cuniculi* at 24 and 48 h post-infection (hpi) (29).

**Fig 1:**
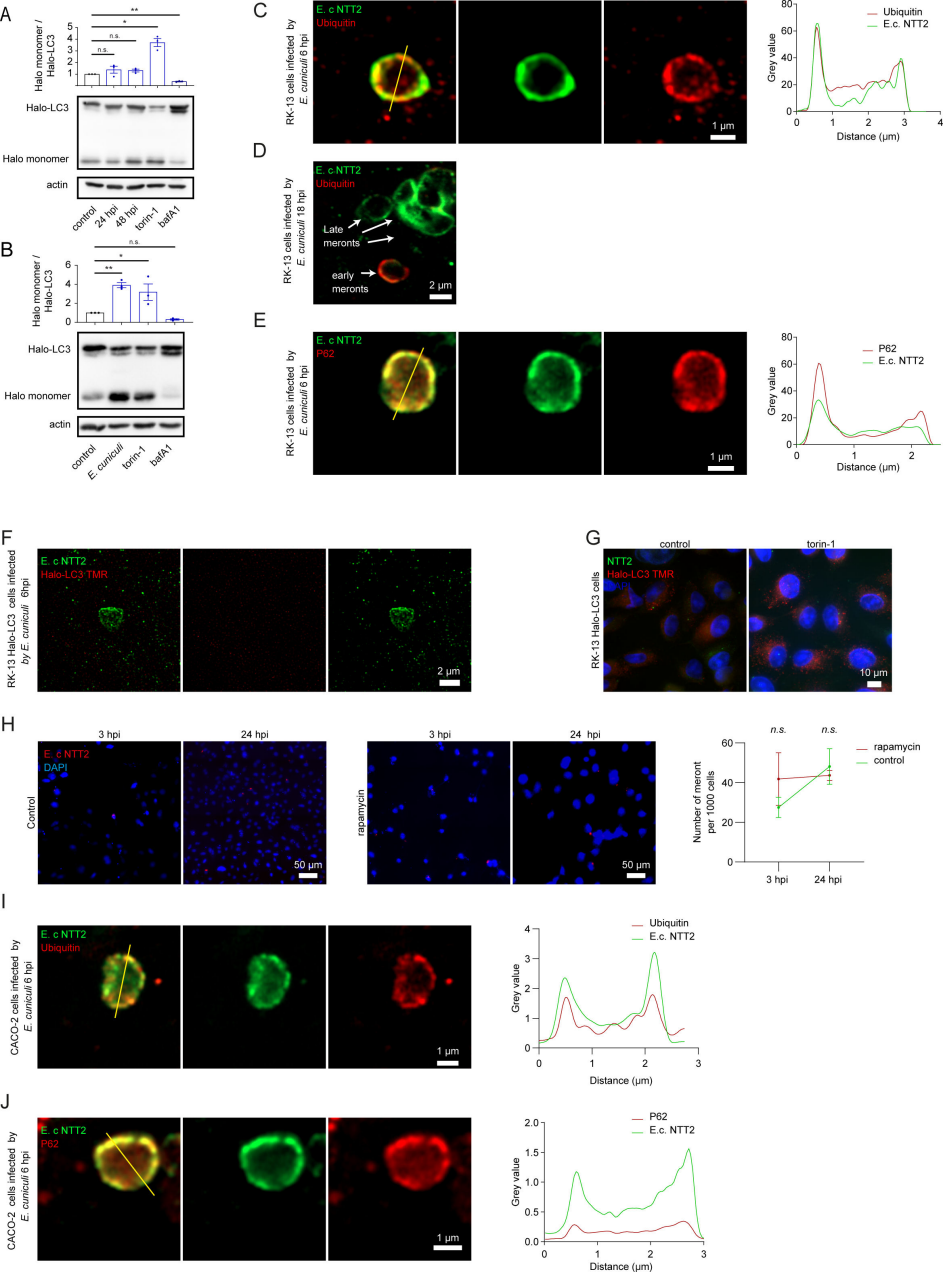
*E. cuniculi* infection increases autophagy flux but is only partially targeted by mammalian host cell autophagy. Immunoblot analyses and quantification of Halo monomer accumulation in RK-13 cells expressing Halo-LC3 and infected with *E. cuniculi* in a time course experiment (**A**) or following a long-term infection (**B**). (**C–E**) Confocal super resolution imaging of RK-13 cells 6 hpi with *E. cuniculi*. Parasite PV is labeled in green with α-E.c.NTT2, and p62 is in red. Yellow lines indicate transects used to generate plots of the intensity value for green and red signals. (**F**) Confocal super resolution imaging of RK-13 Halo-LC3 cells 6 hpi with *E. cuniculi* labeled with TMR Halo ligand. Parasite PV is labeled in green with α-E.c.NTT2 and Halo-LC3 in red. (**G**) Control fluorescence imaging of non-infected RK-13 Halo-LC3 cells treated with rapamycin (250 nM) for 24 h and labeled with Halo ligand TMR. (**H**) Fluorescence imaging of FISH-stained meronts 3 hpi and 24 hpi with or without rapamycin treatment (250 nM) and quantification of infection. Meronts are labeled in red. (**I, J**) Confocal super resolution imaging of CACO-2 cells 48 hpi with *E. cuniculi*. Parasite PV is labeled in green with α-E.c.NTT2, ubiquitin or p62 in red. Yellow lines represent the transects along which the fluorescence intensity of the green and red signals was measured and plotted. In Figure 1A and B, a one-way ANOVA followed by a Sidak’s multiple comparisons test was applied. For figure 1H, a two-way ANOVA followed by a Tukey’s multiple comparisons was used. ***P < 0.001, **P < 0.01, *P < 0.05.

Autophagy flux was assessed using a novel assay involving a Halo-LC3 reporter, which undergoes conversion into free Halo monomers upon autophagy activation (29). By introducing a non-fluorescent Halo ligand into the culture, Halo monomer stabilization in autolysosomes was achieved, with an accumulation of the monomer being quantified via western blot analysis, thus reflecting autophagic flux. To validate this assay, RK-13 cells transduced to stably express Halo-LC3 were treated with increasing concentrations of torin-1 and rapamycin, two potent mTOR inhibitors (30). Both treatments resulted in a clear, dose-dependent induction of the autophagy flux, confirming the sensitivity of the assay (Fig. S1A). Next, Halo-LC3-expressing RK-13 cells were infected with *E. cuniculi*, and autophagic flux was measured at 24 and 48 hpi. At both time points, a slight but non-significant increase in autophagic flux was observed (Fig. 1A). However, we speculated that the low proportion of infected cells might have diluted the observed signal.

To test this hypothesis, we infected RK-13 cells and passaged them several times, achieving a higher infection rate. Under these conditions, autophagic flux was significantly increased in infected cells compared to uninfected controls, confirming the activation of the autophagy pathway (Fig. 1B). These results suggest that microsporidia infection in mammalian cells can upregulate autophagy flux, potentially either activating xenophagy for parasite clearance or facilitating host nutrient diversion by the parasite.

Xenophagy is initiated by the selective labelling of pathogens with ubiquitin, a process mediated by an E3 ubiquitin ligase. Ubiquitylation motifs are recognized by the UBA domain of the receptor protein p62, which, upon oligomerization, recruits the autophagy machinery via its LC3-interacting region (LIR), leading to the encapsulation of the pathogen within an autophagosome (31). If microsporidia are indeed targeted by xenophagy in our system, co-localization of the parasite with ubiquitin, p62, and LC3 should be observed. Notably, previous studies in *C. elegans* demonstrated CUL-6-dependent ubiquitylation of early-stage microsporidia and co-localization with the *C. elegans* LC3/ATG8 orthologue LGG-1 (17).

To assess whether this xenophagic mechanism is conserved in mammalian cells, we investigated the co-localization of p62 and conjugated ubiquitin (FK-2) with *E. cuniculi* meronts. We labeled the parasite using an antibody against E.c.NTT2, a nucleotide transporter identified in the *E. cuniculi* genome (28). This marker was chosen because of its high expression throughout the *E. cuniculi* life cycle, from early meronts to spore formation, and its strong labelling of the PV membrane (Fig. S1B). Similar to the *C. elegans* model, super-resolution fluorescence imaging of early meronts in RK-13 cells detected their labeling by the ubiquitin six hpi (Fig. 1C and D). Intriguingly, only 23% (59/258) parasite stages were ubiquitin-labeled (Fig. 1D), while later stages showed no labeling, suggesting that xenophagy selectively targets early meronts. To further confirm this, we examined the co-localization of p62 and LC3 with the parasite membrane at six hpi. As expected, p62 was present on a large fraction of the parasites (96% - 44/46) (Fig. 1E); however, no co-localization with Halo-LC3 (labeled with TMR) was observed (Fig. 1F). In contrast, torin-1-treated RK-13 cells displayed strong LC3 dot formation as a positive control (Fig. 1G). These results suggest that *E. cuniculi* is targeted by host cell xenophagy but may employ evasion mechanisms to prevent sequestration into fully matured autophagic vesicles, as indicated by the absence of LC3 labeling.

To assess the efficacy of xenophagy in controlling *E. cuniculi* infection, we examined whether the number of infection events decreased between an early (3 hpi) and later (24 hpi) time points. This approach mirrors previous observations in *C. elegans* infected at the L1 stage, where xenophagy reduces pathogen load over time (18). If xenophagy were effective in our model, we would expect a decrease in meronts per cell at 24 hpi compared to 3 hpi, with an enhanced reduction following autophagy induction via rapamycin treatment. To test this hypothesis, RK-13 cells were infected with or without rapamycin treatment, and meronts were stained using FISH at 3and 24 hpi. However, consistent with the lack of LC3 co-localization, no significant differences were observed in the number of infection events between 3 and 24 hpi, with or without rapamycin (Fig. 1H). These results suggest that xenophagy is ineffective at clearing early infection stages of *E. cuniculi* in RK-13 cells.

Since *E. cuniculi* naturally infects a wide range of mammals, predominantly targeting the intestinal epithelium, we sought to validate our findings in a more physiologically relevant system. We performed the same experiments in human colorectal adenocarcinoma cells (CACO-2), a widely used model for studying pathogen interactions with intestinal cells. The results in CACO-2 cells mirrored those in RK-13 cells as early meronts were labeled with both ubiquitin (28% - 17/61) and p62 (91% - 61/67) (Fig. 1I and J).

### Activation of autophagy promotes proliferation of Microsporidia in mammalian cells

**Fig 2:**
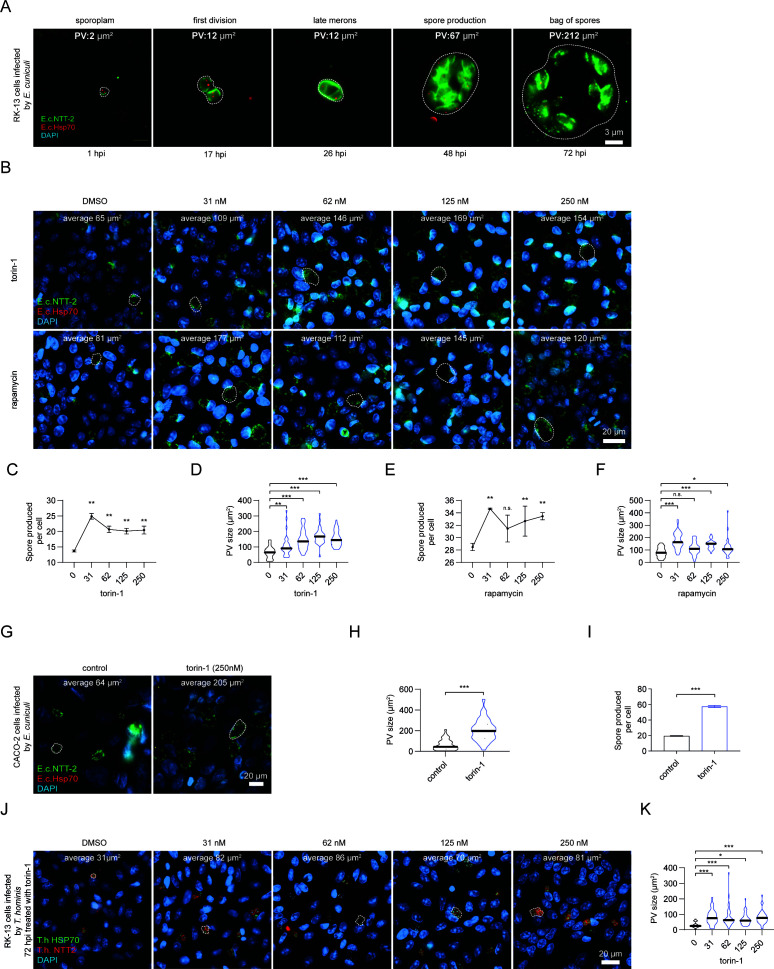
Pharmacological induction of autophagy increases *E. cuniculi* infection in RK-13 and CACO-2 cells. (**A**) Fluorescence imaging of RK-13 cells at different time points post-infection with *E. cuniculi*, from 6 to 72 hpi. Parasite PV is labeled in green with α-E.c.NTT2 and in red with α-HSP70. PV is highlighted by a dashed line and area in µm^2^ measured with ImageJ as indicated on every picture. (**B**) Representative fluorescence imaging of RK-13 cells 48 hpi with *E. cuniculi* in the presence of increasing doses of torin-1 or rapamycin. Parasite PV is labeled in green with α-E.c.NTT2 and in red with α-HSP70. In each picture, a representative PV is highlighted with a dashed line, and the average area of the PV for this condition is indicated. (**C and E**) Graphs indicating the number of *E. cuniculi* spores produced per RK-13 cells 7 dpi with different concentrations of torin-1 or rapamycin. (**D and F**) Graphs showing areas of the PV measured in different treatments. Mean area of the replicates is indicated by the black bar with error bars as SD. At least 30 measurements per condition were performed. (**G**) Representative fluorescence imaging of CACO-2 cells 48 hpi with *E. cuniculi* in the presence of 250 nM torin-1 or control vehicle. Parasite PV is labeled in green with α-E.c.NTT2 and in red with α-HSP70. In each picture, a representative PV is highlighted with a dashed line, and the average area of the PV for this condition is indicated. (**H**) Graph indicating the number of *E. cuniculi* spores produced per CACO-2 cells 7 dpi with 250 nM torin-1 or DMSO. (**I**) Areas of the PV measured in the two different treatments. Mean area of the replicates is indicated by black bar with error bars as SD. At least 30 measurements per condition were performed. (**J**) Representative fluorescence imaging of RK-13 cells 72 hpi by *T. hominis* in the presence of increasing doses of torin-1. Parasite PV is labeled in red with α-T.h.NTT4 and in green with α-HSP70. In each picture, a representative PV is highlighted with a dashed line, and the average area of the PV for each condition is indicated. (**K**) Graph showing areas of the PV measured in different treatments. Mean area of the replicates is indicated by the black bar with error bars as SD. In figure 2C-F and K, a one-way ANOVA followed by a Sidak’s multiple comparisons test was applied. For figure H and I, a paired two-tailed Student’s *t* -test was used. ***P < 0.001, **P < 0.01, *P < 0.05.

Since xenophagy does not appear to clear *E. cuniculi* infection in mammalian cells, we next examined whether modulation of the autophagy pathway affects parasite infection progression. To measure *E. cuniculi* proliferation, we used two metrics: spore production per host cell 7 days post-infection (dpi) and the size of the PV at 48 hpi. The 48 hpi time point was chosen because it marks the transition between the parasite’s initial growth phase—where the PV forms and the parasite begins to divide—and the beginning of spore production, which occurs at the PV centre, with newly formed meronts accumulating at the periphery (Fig. 2A). Thus, PV size serves as a reliable proxy for parasite proliferation.

To induce autophagy, we treated cells with torin-1. Given that torin-1 can also induce cell cycle arrest and protein synthesis inhibition, we also tested rapamycin, a selective mTORC1 inhibitor, to confirm that observed effects were due to autophagy induction (32).

**Fig 3:**
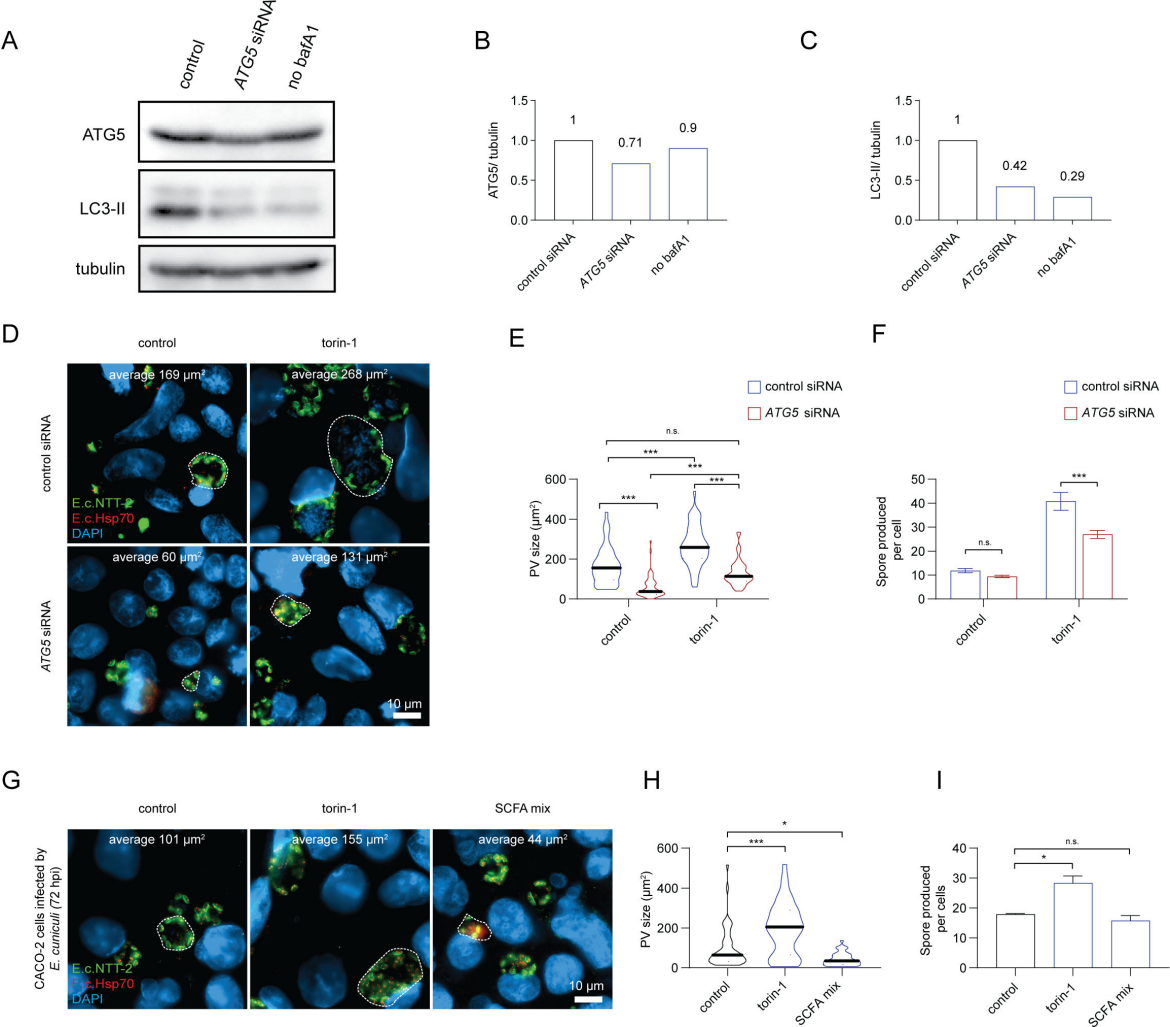
Reduction of autophagic flux by ATG5 silencing and SCFA treatment limits *E. cuniculi* proliferation in host cells. (**A**) Western blot of LC3-II, ATG5 and tubulin (loading control) after *ATG5* siRNA treatment of CACO-2 cells. (**B**) Quantification of ATG5 abundance from western blot shown in (A). (**C**) Quantification of LC3-II accumulation shown in western blot. (**D**) Representative fluorescence imaging of RK-13 cells 48 hpi with *E. cuniculi* in the presence or absence of torin-1 (250 nM) and with or without *ATG5* siRNA treatment. In each image, a representative PV is highlighted with a dashed line, and the average area of the PV for this condition is indicated. (**E**) Graph showing areas of the PV measured in the different treatments. Mean area of the replicates is indicated by black bar with error bars as SD. (**F**) Graph indicating the number of *E. cuniculi* spores produced per RK-13 cell 7 dpi with different treatments. (**G**) Representative fluorescence imaging of RK-13 cells 48 hpi with *E. cuniculi* in the presence of 250 nM torin-1, SCFA mix or PBS. In each picture, a representative PV is highlighted with a dashed line, and the average area of the PV for this condition is indicated. (**H**) Graph showing areas of the PV measured in the different treatments. Mean area of the replicates is indicated by black bar with error bars as SD. (**I**) Graph indicating the number of *E. cuniculi* spores produced per RK-13 cell 7 dpi with the different treatments. Three independent biological replicates per point, error bars indicate SD. In figure E and F, a 2two-way ANOVA followed by a Tukey’s multiple comparisons was applied. for figure H and I, we used a one-way ANOVA followed by Sidak’s multiple comparisons test. ***P < 0.001, **P < 0.01, *P < 0.05.

Surprisingly, rather than inhibiting parasite proliferation, both torin-1 and rapamycin enhanced it. With torin-1, spore production at 7 dpi significantly increased, reaching a peak at 31 nM, where spore numbers doubled compared to DMSO-treated controls (Fig. 2B and C). PV size also increased with torin-1 treatment, peaking at 125 nM (Fig. 2D). Rapamycin treatment produced similar, though more moderate, effects, increasing both spore production (Fig. 2B and E) and PV size (Fig. 3F) at all doses except 62 nM. These findings suggest that autophagy induction benefits parasite proliferation rather than suppressing it, further supporting our earlier observation that *E. cuniculi* is not degraded by xenophagy. The differing effects between Torin-1 and rapamycin on parasite growth may reflect their differential potency in inhibiting mTOR (30).

To investigate whether the parasite’s ability to exploit host autophagy is conserved across cell types, we repeated these experiments using CACO-2 cells. Treatment with the maximum dose of torin-1 (250 nM) led to even greater increases in both spore production (300% compared to controls) and PV size at 48 hpi, indicating that *E. cuniculi* benefits from autophagy induction in this intestinal epithelial cell model as well (Fig. 2G through I).

We also tested whether other Microsporidian species that infect mammals can similarly exploit host autophagy. We examined the effect of torin-1 on *Trachiopleistophora hominis*, a species isolated from HIV patients (33, 34). Using an antibody against *T. hominis* NTT4 (a nucleotide transporter highly expressed throughout its lifecycle), we found that torin-1 treatment significantly increased PV size, similar to the results observed with *E. cuniculi* (Fig. 2J and K).

Collectively, these results suggest that autophagy activation in the host cell may promote the proliferation of both *E. cuniculi* and *T. hominis*, as evidenced by the increased PV size and spore production. This supports our hypothesis that *E. cuniculi* has evolved mechanisms to evade xenophagy and instead exploit autophagy to acquire nutrients from the host. The fact that this phenomenon was observed in two different cell lines and two distantly related Microsporidian species suggests that it may be a conserved feature among these parasites.

### Suppression of autophagy drastically reduces *E. cuniculi* proliferation

While induction of autophagy with torin-1 and rapamycin is a robust and well-characterized approach, these drugs can also trigger adverse effects, such as cell cycle arrest, inhibition of protein synthesis, and cell death at higher doses (35). To validate our findings and eliminate the potential confounding effects of these treatments, we investigated the impact of autophagy inhibition on *E. cuniculi* proliferation using siRNA targeting *ATG5*, a key autophagy gene, in the presence or absence of torin-1. This experiment was conducted in CACO-2 cells to take advantage of reagents optimized for human cells.

To confirm *ATG5* knockdown efficiency, we assessed ATG5 protein levels and LC3-II accumulation following bafilomycin A1 treatment by immunoblotting in cells transfected with *ATG5* or non-targeting siRNA. Despite a modest reduction in the ATG5 protein levels, knockdown resulted in a 58% decrease in LC3-II accumulation after bafilomycin A1 treatment, indicating a significant reduction in autophagy flux (Fig. 3A through C).

Confocal microscopy of CACO-2 cells 48 hpi revealed that *ATG5* silencing significantly reduced both the size of the *E. cuniculi* PV and the number of spores produced per cell, in both torin-1 and DMSO-treated conditions (Fig. 3D through F). Additionally, *ATG5* knockdown reversed the increase in PV area seen with torin-1 treatment, supporting the hypothesis that the enhanced parasite proliferation and PV size observed with torin-1 is dependent on autophagy induction. While *ATG5* knockdown also suppressed the increase in spore production following torin-1 treatment, this effect was partial (Fig. 3F). This may be due to the measurement being taken at 7 dpi, a time point when siRNA efficacy could begin to diminish.

Our findings suggest that disturbances in autophagy flux, whether due to genetic factors (such as those associated with CD or IBD) or environmental influences (such as changes in the gut microbiota), may influence susceptibility to Microsporidian infection. Notably, a previous study reported a higher prevalence of Microsporidian infections in CD patients, particularly with *E. cuniculi*, implying a potential increased susceptibility in these individuals (14).

Microbial metabolites, such as short-chain fatty acids (SCFAs), can significantly impact gut epithelial cell metabolism and physiology. For instance, exposure of CACO-2 cells to SCFAs—by-products of the intestinal microbiota’s fermentation of dietary fibers—has been linked to a reduction in autophagy flux and an enhancement of epithelial barrier function, both of which may confer protection against Microsporidian infections (36). To explore this further, we investigated whether modulating autophagy flux through SCFA treatment could influence *E. cuniculi* proliferation in CACO-2 cells.

For this experiment, CACO-2 cells were pre-treated with a SCFA mix (0.5 mM acetate, 0.01 mM butyrate, and 0.01 mM propionate), following the protocol in reference 36 for 48 h prior to infection. SCFA treatment was maintained throughout the experiment. Our results showed a significant reduction in the size of the PV at 48 hpi in SCFA-treated cells compared to controls (Fig. 3G and H). While there was a trend toward reduced spore production per cell in the SCFA-treated group, this difference did not reach statistical significance (Fig. 3I).

## DISCUSSION

Autophagy is a key cellular mechanism activated in response to cytoplasmic stress, nutrient deprivation, and protein aggregation, all of which can result from intracellular pathogen infections. However, the role of autophagy in mammalian cell responses to Microsporidian infections remains underexplored.

In this study, we demonstrate that *E. cuniculi* infection robustly induces autophagy in RK-13 cells, with early-stage meronts partially marked by host autophagy-related proteins, ubiquitin and p62. Interestingly, despite this labeling, *E. cuniculi* evades engulfment by LC3-decorated autophagosomes, and no reduction in infection rates was observed between 3 and 24 hpi. These findings suggest that *E. cuniculi* effectively escapes xenophagy and avoids degradation. Beyond evasion, our data also indicate that the parasite exploits host autophagy as a nutrient source, as evidenced by the significant increase in parasite proliferation following pharmacological induction of autophagy using torin-1 or rapamycin. This exploitation of autophagy appears to be conserved across Microsporidia species, as *T. hominis* exhibited a similar response. Knockdown experiments using *ATG*5 siRNA further confirmed that suppression of host autophagy limits *E. cuniculi* proliferation, highlighting the dependency of the parasite on the autophagy pathway for successful proliferation in human cells.

Furthermore, we investigated the role of microbiota-derived secondary metabolites, particularly SCFAs, in modulating host autophagy and their potential impact on *E. cuniculi* infection. Our findings demonstrated that SCFA treatment significantly reduced *E. cuniculi* proliferation in CACO-2 cells, which we hypothesize is due to SCFAs' modulation of autophagy flux. This supports the notion that perturbations in autophagy flux, whether through genetic predispositions (such as CD or IBD) or environmental influences (e.g., microbiota metabolites), may influence susceptibility to Microsporidian infection.

We propose that, in contrast to *C. elegans*, where autophagy plays a role in controlling Microsporidian infections (17, 18), eucaryotic parasites have evolved strategies to evade and exploit autophagy of the mammalian host cell. Our findings suggest that *E. cuniculi* avoids xenophagy, despite ubiquitin and p62 labeling, which indicates an active evasion mechanism requiring further investigation. A similar phenomenon has been observed in *P. berghei* infections, where xenophagy suppression promotes parasite survival. However, unlike *Plasmodium*, where xenophagy limits parasite proliferation, our results suggest that xenophagy is ineffective in controlling *E. cuniculi* infection in mammalian cells. Knockdown of ATG5 in human cells led to a reduction in both the PV size and parasite proliferation, highlighting the reliance of *E. cuniculi* on host autophagy for growth.

The autophagy pathway is implicated in various pathologies, including IBD (37), which has been associated with Microsporidian infections (14). A meta-analysis using the IBD-TaMMA platform (38) identified four Microsporidia species, including *E. cuniculi*, as significantly more abundant in stool samples from CD patients compared to healthy controls (Table S1). This association may be influenced by factors such as immunosuppressive treatments commonly administered to IBD patients, which could facilitate Microsporidian infection (39). Microsporidia are increasingly recognized as ubiquitous pathogens, capable of infecting a wide range of hosts, including humans (11). Recent studies have identified a high prevalence (14.9%) of *E. bieneusi* infections in children with IBD undergoing immunosuppressive therapy (40). The compromised epithelial barrier function characteristic of IBD (41) may also contribute to increased susceptibility to Microsporidian infections.

Our results show that perturbations in the autophagy flux within intestinal epithelial cells can directly impact Microsporidian proliferation. Conversely, by interfering with host autophagy and epithelial barrier function, Microsporidia could potentially act as triggers for IBD or exacerbate its progression. Thus, Microsporidia may play a dual role in both the initiation and worsening of IBD. These findings underscore the need for further investigation into the role of Microsporidia in IBD, which could improve patient stratification and lead to better clinical outcomes in managing this highly heterogeneous disease.

In addition to these clinical implications, our work provides broader insight into the evolution of host-pathogen interactions in Microsporidia. Despite their vast biological differences, *E. cuniculi* and *T. hominis*—two evolutionarily distant Microsporidian species infecting the same host cell types—both subvert host autophagy to overcome their extreme metabolic reduction and support intracellular proliferation. These species differ markedly in genome content, susceptibility to antimicrosporidian drugs, and strategies for cell division and development. Yet, both appear to rely on host autophagy as a nutrient source. Together with earlier findings from nematode-infecting Microsporidia, our results suggest that subverting host autophagy is a deeply conserved and fundamental adaptation across Microsporidia lineages. This strategy likely represents one of the core innovations that enabled the emergence of their obligate intracellular lifestyle.

## MATERIALS AND METHODS

### Cell and parasite culture

RK-13 rabbit kidney cell line obtained from the ATCC (ATCC CCL-37) was grown at 37°C with 5% CO_2_ in Dulbecco’s modified Eagle medium (DMEM) (Gibco), containing 10% FBS (Gibco), penicillin (100 µg/mL), streptomycin (100 µg/mL), kanamycin (100 µg/mL), and Amphotericin B (1 µg/mL) purchased from Sigma. CACO-2 cell line obtained from ATCC (ATCC HTB-37) was grown at 37°C in complete modified Eagle medium (MEM) medium complemented with 20% FBS, penicillin (100 µg/mL), streptomycin (100 µg/mL), kanamycin (100 µg/mL), and Amphotericin B (1 µg/mL). The Microsporidia *E. cuniculi* EC2 and *T. hominis* (ATCC PRA-404) were routinely subcultured in RK-13 cells grown as previously described (28). Spores used in time course experiments were harvested from infected RK-13 cells grown in 175 cm^2^ flasks. Cells were washed and scraped in phosphate-buffered saline (PBS) and then lysed by three passages through a 25G needle and three rounds of 1 min sonication on ice. Released spores were purified by layering onto a 25% Percoll gradient and centrifugation at 900 × *g* for 30 min. After three washes in PBS, the pelleted spores were resuspended in PBS and used within a week to infect RK-13 or CACO-2 cells.

### Generation of stable cell lines

Generation of cells stably expressing Halo-LC3 constructs was achieved by packaging retroviruses in the HEK293FT (293FT) cell line. The cells were seeded in a 10 cm dish (6.0 × 10^6^ cells/10 mL/dish) in antibiotic-free culture medium. The next day, the cells were transfected with plasmids containing the packaging gag/pol (retrovirus) or psPAX2 (lentivirus) and envelope pCMV-VSV-G genes, pMRX-IP-HaloTag7-LC3 (gift from Noboru Mizushima, Addgene, 184901)(42) using Lipofectamine 3000. Following overnight transfection, the medium was replaced with fresh antibiotic-free medium. After 48 h virus-containing medium was collected, and viruses were concentrated using LentiX concentrator reagent (Takara Bio). RK-13 cells were then transduced using concentrated virus and selected after 24 h using 8 µg/mL of puromycin (Invitrogen).

### Time course experiments

RK-13 cells or CACO-2 cells were seeded into 24-well (for protein extraction) or 6-well plates (for immunofluorescence and spore production assays). When confluent, purified spores were added with a multiplicity of infection of 200. After 30 min, the cells were washed two times with medium, and fresh medium with or without treatment was added (torin-1 31–250 nM *Selleck Chemicals*, rapamycin 12.5–250 nM *Selleck Chemicals*, SCFA mix [acetate 0.5 mM, butyrate 0.01 mM, and propionate 0.01 mM] *Sigma-Aldrich*, DMSO, PBS). Medium was changed every 3 days and treatment was maintained for the full duration of the experiment.

### Spore production assay

At 7 dpi, the cells were washed two times with PBS and detached with trypsin. Part of the cell suspension was used to quantify the number of cells per well. The remaining suspension was centrifuged for 5 min at 16,000 × *g*. The pellet was resuspended in RIPA buffer with 1% SDS. The lysate was sonicated for 1 min at 4°C to reduce the viscosity and centrifuged for 5 min at 16,000 × *g*. The pelleted spores were then resuspended in PBS and counted on a Neubauer counting chamber. All assays were done in three independent biological triplicates. Spores produced per cell were then calculated and plotted in GraphPad Prism.

### Autophagy flux measurement by immunoblotting

Long-term infected RK-13 cells expressing Halo-LC3 or RK-13 cells infected through a kinetic of infection protocol were treated with either DMSO, 100 nM rapamycin, 400 nM bafilomycin A1 (Enzo Life Sciences), and 20 µM 7-Bromo-1-heptanol (HaloTag-blocking agent) (Thermo Scientific Chemicals) (43) for 24 h before protein extraction. To test the effect of torin-1 and rapamycin on autophagy flux, RK-13 cells expressing Halo-LC3 were treated with the following compounds: rapamycin (12.5–250 nM), torin-1 (12.5–250 nM), bafilomycin A1 400 nM, and 20 µM 7-Bromo-1-heptanol for 24 h before protein extraction.

### Immunofluorescence assays

At 6, 48, or 72 hpi, coverslips were washed three times with PBS and fixed for 15 min in ice-cold 50% methanol, 50% acetone. The coverslips were later rehydrated in PBS for 15 min, blocked in 3% fat-free dry milk in PBS, and labeled with primary antibodies diluted in blocking buffer overnight at 4°C in a humid chamber. Mouse α-conjugated ubiquitin (1:100; Enzo Life Sciences), mouse α-p62 (1:100; Cell Signaling Technology), rat α-E.c.NTT2 (1:200; [28]), rabbit α-E.c.HSP70 (1:500; [28]), rabbit α-T.h.NTT4 (1:200; [29]), and rat α-T.h.HSP70 (1:500; [29]). Then labeled for 2 h at room temperature with secondary antibodies (α-rat Alexa fluor 488, α-rabbit Alexa fluor 568, α-rabbit Alexa fluor 594; *Invitrogen*), counterstained with DAPI and mounted on slides with Prolong glass antifade mounting solution. For co-localization assays, the slides were imaged with a 63× magnification on a Zeiss LSM800 confocal microscope using Airyscan super-resolution mode. The red and green signals were then quantified over a transect using ImageJ, and intensity values were plotted using GraphPad Prism. For kinetic of infection experiments, the slides were imaged using a Zeiss AxioImager with a 63× magnification. For each condition, images were taken randomly on three slides using the same acquisition parameters across each set of experiments. The images were then blinded using a home-made Python script, and the measurement of the PVs was done using a home-made ImageJ macro. For each condition and experiment, at least 30 PVs were measured.

### FISH staining

For quantification of infection with or without rapamycin 250 nM treatment, RK-13 cells were infected using the time course protocol, and (3 and 24 hpi), the culture medium was removed, and cells were fixed in Farmer’s fixative (1/4 glacial acetic acid and 3/4 absolute ethanol) for 2 h at 4°C. After two washes of 15 min in PBS, a pre-hybridization was done in a solution PBS:hybridization buffer (TH) containing 20 mM Tris-HCl (pH 7.8), 0.9 M NaCl, 1 × Denhardt’s solution (VWR Life Science), and 0.01% SDS for 15 min at room temperature, followed by two incubations in TH solution, one for 15 min at room temperature, and one at 45°C for 20 min. Eventually, the TH solution was replaced by the hybridization solution containing 50 µM of a 5′-Cy3-labeled ssuRNA *E. cuniculi* specific-probe (Ec01, 5′-CCACAGGGGCAGACCACTAT-3) (44). Hybridization was performed for 2H30 at 45°C and was followed by four 20 min washes with warmed TH solution, one 15 min wash with TH:PBS, and eventually replaced with PBS. DNA and chitin were stained with DAPI DY96 respectively overnight. Coverslips were mounted with Prolong Diamond Antifade Mounting. The percentage of infected cells was assessed by counting cell nuclei and infectious foci in 11 randomly chosen fields from each coverslip.

### Immunoblotting

Protein concentration was measured using the DC Protein Assay (BioRad) and a FLUOstar Omega plate reader (BMG Labtech). Samples were prepared by boiling in SDS Loading buffer (BioRad) at 100°C for 5 min in the presence or absence of 2.5% β-ME (β-mercaptoethanol, Sigma). 20 µg of protein was run on 10% Tris-Glycine SDS-PAGE gels and transferred to Immobilon-P (Millipore) PVDF membranes. Blots were blocked for 1 h (PBS containing 5% fat-free dry milk and 0.1% Tween-20) and incubated with primary antibodies diluted in blocking solution at 4°C overnight: Mouse α-Halo 1:1,000 (Promega, #G9211), rabbit α-LC3B 1:1,000 (Cell Signaling Technology), rabbit α-Atg5 1:1,000 (Cell Signalling Technology), mouse α-tubulin 1:1,000 (Sigma Aldrich), and mouse anti-β-actin 1:5,000 (St John’s Laboratory). Then, blots were incubated with secondary HRP-conjugated antibodies α-mouse 1:5,000 (Sigma Aldrich) or α-rabbit HRP 1:5,000 (Sigma Aldrich) in blocking solution for 45 min at room temperature and revealed for 5 min with the Clarity Western ECL Substrate (BioRad). Signals were detected by chemiluminescence using an iBright Imaging Systems (ThermoFisher Scientific). Autophagic activity was then quantified as a ratio of Halo monomer per Halo-GFP-LC3 using Fiji/ImageJ (version 1.54i; NIH).

### siRNA transfection

ON-TARGETplus SMARTpool siRNA against human *ATG5* (L-004374-00-0005) and non-targeting SMARTpool siRNA (D-001810-10-05) were purchased from *Horizon Discovery*. Silencing was done in two steps. A first transfection with a final siRNA concentration of 100 nM was done for 24 h in a six-well plate, followed by a passaging of the transfected cells into 24-well plates. Twenty-four hours after plating, the cells were transfected a second time with a final concentration of 20 nM siRNA. Twenty-four hours after the second treatment, the cells were infected and treated with torin-1 (250 nM) or DMSO as a control.

### Statistical analyses

GraphPad Prism version 10.00 was used for statistical analyses and statistical presentation of quantitation. *P* values are presented in the figures above the compared conditions: ***P < 0.001, **P < 0.01, *P < 0.05. Where two conditions were compared, a paired two-tailed Student’s *t*-test was used. If more than two conditions were compared, a one-way ANOVA followed by Sidak’s multiple comparisons test was applied. For grouped data, a two-way ANOVA followed by Tukey’s multiple comparisons was applied.

## SUPPLEMENTAL MATERIAL

Supplemental material mbio.01049-25-s0001.pdf Table S1 and Figure S1.

ASM does not own the copyrights to Supplemental Material that may be linked to, or accessed through, an article. The authors have granted ASM a non-exclusive, world-wide license to publish the Supplemental Material files. Please contact the corresponding author directly for reuse.
