# Supplementary material for: Microsporidian obligate intracellular parasites subvert autophagy of infected mammalian cells to promote their own growth
Source: mBio. 2025 May 30;16(7):e01049-25. doi: 10.1128/mbio.01049-25 (PMC12239571; doi:10.1128/mbio.01049-25)
Supplement: Supplemental material — Table S1 and Figure S1. [file mbio.01049-25-s0001.pdf]

Supplemetary Figure 1

A

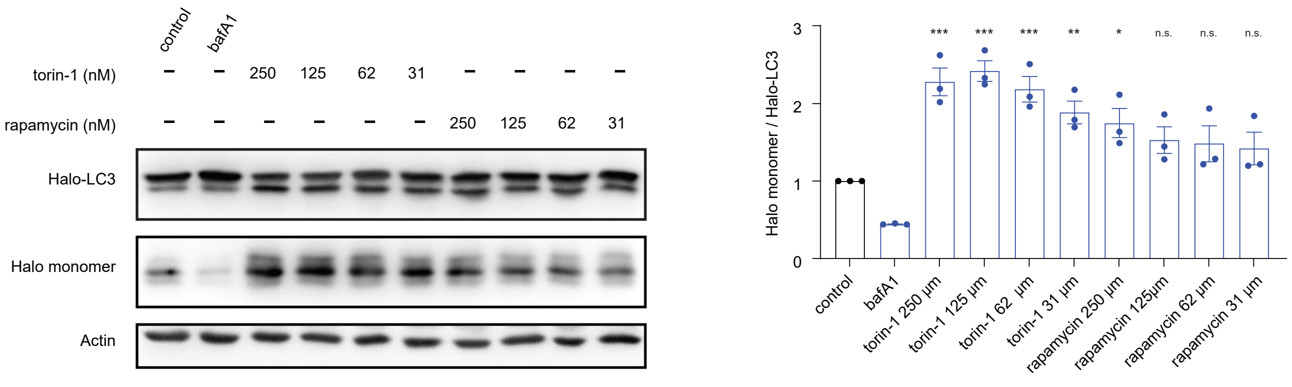

B

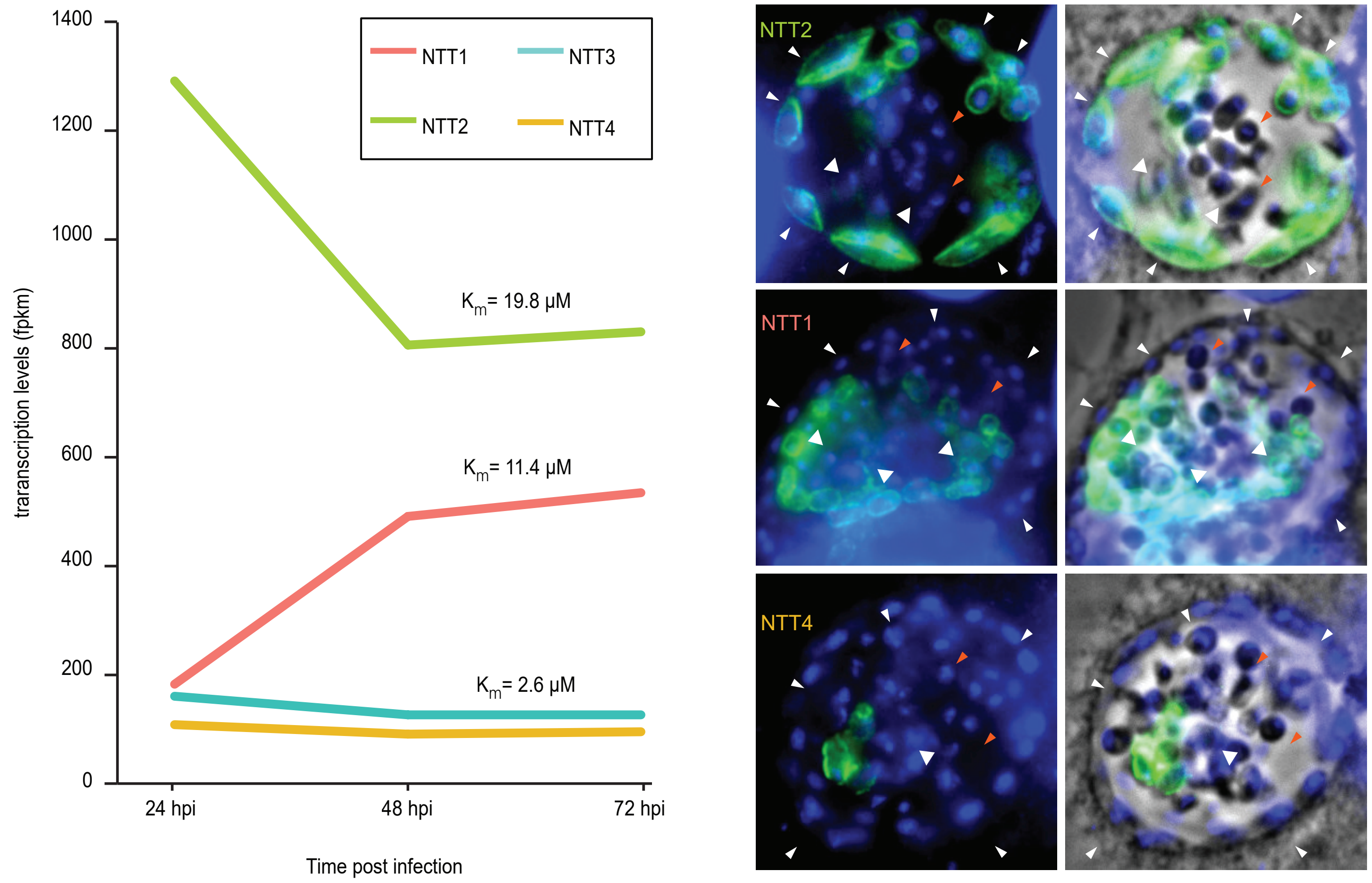

Supplementary Figure 1. (A) Immunoblotting analyses of autophagy flux in RK-13 cells treated with increasing doses of rapamycin or torin-1. Mean of the replicates is plotted with errors bars as SD. Each dot represents one measure and significance against control is shown. (B) NTT2 transporter is expressed during the whole length of E. cuniculi life cycle by meronts localised on the edge of the PV. Expression level of NTTs transcript during the E. cuniculi time course experiment (data from Grisdale et al., 2013). Km for each of the nucleotide transporter are indicated on the graph (Tsaousis et al., 2008). Early meronts labelled with NTT1, NTT2 or NTT4 antibodies. NTT2 antibodies label the periphery of the PV alongside the membrane (white arrowheads). Sporonts (white arrowhead) labelled with NTT3 antibodies were localized closer to the centre of the vesicle. NTT4 antibodies label small sub-population of cells observed in large PVs corresponding to the late stage of infection but not in any of the time points in the time course experiment. Spores (orange arrowheads) were not labelled with any of the antibodies tested

Supplemetary Table 1

**Species**	**log2 FC**	**log2 FC SE**	**P-value**	**FDR**	**Average abundance**
Malassezia restricta	4.57535756	0.17844045	5.35E-145	3.05E-143	182.211187
Thermothelomyces thermophilus	0.98303243	0.1198278	2.33E-16	3.32E-15	4.52157841
Candida albicans	1.03910564	0.14806304	2.25E-12	2.14E-11	3.58746262
Pyricularia pennisetigena	0.70320105	0.10881108	1.03E-10	8.38E-10	1.86912502
Lachancea thermotolerans	0.63259641	0.1160302	4.98E-08	2.84E-07	3.09992751
Yarrowia lipolytica	0.60830223	0.12108713	5.07E-07	2.63E-06	5.27013171
Thermothielavioides terrestris	0.50519154	0.12294842	3.97E-05	1.62E-04	7.7545517
Fusarium graminearum	0.44209296	0.10963581	5.52E-05	2.10E-04	2.0674997
Encephalitozoon cuniculi	0.52452688	0.14009181	1.81E-04	5.87E-04	1.49142983
Encephalitozoon romaleae	0.50836781	0.13599327	1.85E-04	5.87E-04	1.42695939
Encephalitozoon intestinalis	0.49279246	0.1351326	2.66E-04	7.82E-04	1.4478781
Scheffersomyces stipitis	0.4182443	0.1281762	1.10E-03	2.86E-03	3.70861159
Encephalitozoon hellem	0.43944822	0.13757536	1.40E-03	3.47E-03	1.53669599
Debaryomyces hansenii	0.38680148	0.12229186	1.56E-03	3.71E-03	2.19098728
Ustilago maydis	0.36473875	0.11877212	2.13E-03	4.87E-03	3.52710176
Pyricularia oryzae	0.32144826	0.10913016	3.22E-03	7.07E-03	2.04133019

Supplementary Table 1. Table showing the microbial eukaryote (limited to Fungi) species significantly more abundant (FDR<0.01) in stool-associated tissues from CD patient vs control patient. Microsporidia species are highlighted in red. Data table exported from the IBD Transcriptome and Metatranscriptome Meta-Analysis (IBD-TaMMA) (Massimino et al., 2021) platform that combine data from 26 independent human metatranscriptomics studies with a focus on IBD patients. IBD-TaMMA is accessible through web interface: https://ibd-tamma.readthedocs.io/.
